# Documenting manifestations and impacts of autosomal recessive spastic ataxia of Charlevoix–Saguenay to develop patient-reported outcome

**DOI:** 10.1186/s13023-022-02497-1

**Published:** 2022-10-01

**Authors:** Marjolaine Tremblay, Laura Girard-Côté, Bernard Brais, Cynthia Gagnon

**Affiliations:** 1 Université de Sherbrooke, 2500, boulevard de l’Université, Sherbrooke, QC J1K 2R1 Canada; 2 Groupe de recherche interdisciplinaire sur les maladies neuromusculaires, 2230 de l’Hôpital cp 1200, Jonquière, QC G7X 7X2 Canada; 3 Université du Québec à Chicoutimi, 555, boulevard de l’Université, Chicoutimi, QC G7H 2B1 Canada; 4 McGill University, 845 Sherbrooke Street West, Montréal, QC H3A 0G4 Canada; 5 Montreal Neurological Institute and Hospital, 3801 University Street, Montreal, QC H3A 2B4 Canada; 6 Centre de recherche du Centre hospitalier universitaire de Sherbrooke, 3001, 12e Avenue Nord, aile 9, porte 6, Sherbrooke, QC J1H 5N4 Canada

**Keywords:** Ataxia, Movement disorders, Patient-reported, Phenotype

## Abstract

**Background:**

Autosomal recessive cerebellar ataxias (ARCA) are a group of rare inherited disorders characterized by degeneration or abnormal development of the cerebellum. Autosomal recessive spastic ataxia of Charlevoix–Saguenay (ARSACS) is one of the most prevalent in Europe.

**Objectives:**

The aim of this study is to provide a better understanding of the manifestations and impacts of ARSACS.

**Methods:**

A systematic review of the literature was conducted, followed by a qualitative study using semistructured interviews and discussion groups to obtain the experience of people affected.

**Results:**

According to the PROMIS framework, the results show manifestations and impacts in three components of health: physical, mental, and social. Fatigue and struggles with balance and dexterity are the physical manifestations of the disease most often cited by participants. Negative affects such as frustration and depression are among the mental health impacts with some loss in cognitive abilities. Social health is the least documented component; nonetheless, people with the disease report significant impacts in terms of social relationships, activities and work.

**Conclusions:**

These findings shed new light on the experience of people with recessive ataxia and identify key aspects to assess to improve their overall health.

## Introduction

Autosomal recessive cerebellar ataxias (ARCAs) are a group of rare inherited disorders. This heterogeneous group of disorders is characterized by degeneration or abnormal development of the cerebellum and spinal cord that lead to neurological dysfunctions. The manifestations usually start before the age of 40, but in some cases, they can appear before 20 [98, 126]. The most frequent ARCA is Friedreich ataxia (FA), but there are many other ARCAs, some with higher regional prevalence rates, such as the autosomal recessive spastic ataxia of Charlevoix–Saguenay (ARSACS). ARSACS, originally described in 1978, is a progressive neurological disorder mostly encountered in Québec (Canada) with a prevalence of 1/1 932 in the Charlevoix and Saguenay–Lac-St-Jean regions [13, 32]. The worldwide prevalence is still unknown, but it is estimated to be one of the most common ARCA in Europe, the first being FA [127].

The disorder is caused by a mutation on the *SACS* gene on chromosome 13q12 [41]. ARSACS is characterized by cerebellar (incoordination), neuropathic (distal strength loss and sensation), and pyramidal (spasticity and weakness) manifestations [12]. Disease onset is approximately 3.41 ± 1.55 among individuals with the most frequent mutations in Québec [38]. Lower limb dysfunction and gait restriction become more obvious in the teens, leading to wheelchair use on average approximately 40 years but with a large variability [75]. Even if the clinical progression of ARSACS is thought to be relatively slow, a decrease in mobility, balance and lower limb performance can be documented by clinical outcome measures during a 2-year period [46, 48, 49]. The presentation of the disease is variable and can include upper limb dysfunctions (impaired coordination and dexterity), dysarthria and dysphagia, among others [12, 13, 47]. Social performance and the realization of daily activities are highly impaired in most cases [88].

There is no treatment available to cure ARSACS, but present active research may lead to clinical trials in the near future [73]. Thus, it is essential to be prepared for these clinical trials, and according to the Food and Drug Administration (FDA), a crucial step is the selection of accurate outcome measures to assess how patients feel and function [43]. Patient-reported outcomes (PROs) are a type of outcome measure that are requested by regulatory agencies such as the FDA for use in clinical trials as primary or secondary endpoints. PRO can be defined as “any report of the status of a patient’s health condition that comes directly from the patient, without interpretation of the patient’s response by a clinician or anyone else” ([42], p. 2). PROs are increasingly used as complementary measurements to reflect clinical manifestations of the disease that objective scales cannot observe and establish the impacts of these manifestations on everyday life [65]. At this time, the only published PRO is for ataxias in general [118]. The first step to achieve PRO development for a specific condition is to generate the items by qualitative methods to gather information about the concepts to be measured [25]. Once more, there is no qualitative study that underlines the complete experience of people affected by recessive ataxia, from the clinical manifestations to the impacts on their daily living. Therefore, this study aims to document the manifestations and impacts of the disease according to the perception of people with ARSACS.

## Method

This study used a descriptive qualitative design to reach a deep understanding of people’s affected experiences [50]. To ensure a triangulation of the perspectives, three data collection methods were used: a systematic review of the literature, semistructured interviews, and discussion groups [86]. For the three methods, a conceptual framework was used to develop a blueprint to analyze the data. This framework, the *Patient-Reported Outcomes Measurement Information System* (PROMIS), has been widely used in PRO questionnaires [26]. The PROMIS framework includes three domains of health: physical, mental and social. Each domain has subdomains that have been used to develop the blueprint applied for the data analysis. The blueprint ensures good coverage of the concepts measured and, therefore, helps to support content validity [125].

Since the data collected were used to develop a PRO questionnaire, the FDA guidelines were also followed [42]. These guidelines recommend the inclusion of people affected by the condition in the development process of the PRO. Then, a committee of four persons with ARSACS was established (three from Canada, one from France). These persons are patient research partners and were included significantly in the study as full members of the research team [33]. The academic research team provided basic training in research to the committee when needed. The study was approved by the Ethics Review Board of the *Centre intégré universitaire de santé et de services sociaux du Saguenay–Lac-Saint-Jean* (CIUSSS-SLSJ) and the *Centre intégré universitaire de santé et de services sociaux de la Capitale-Nationale* (Québec, Canada), and informed consent was obtained from all participants.

### Systematic review of the literature

**Fig. 1:**
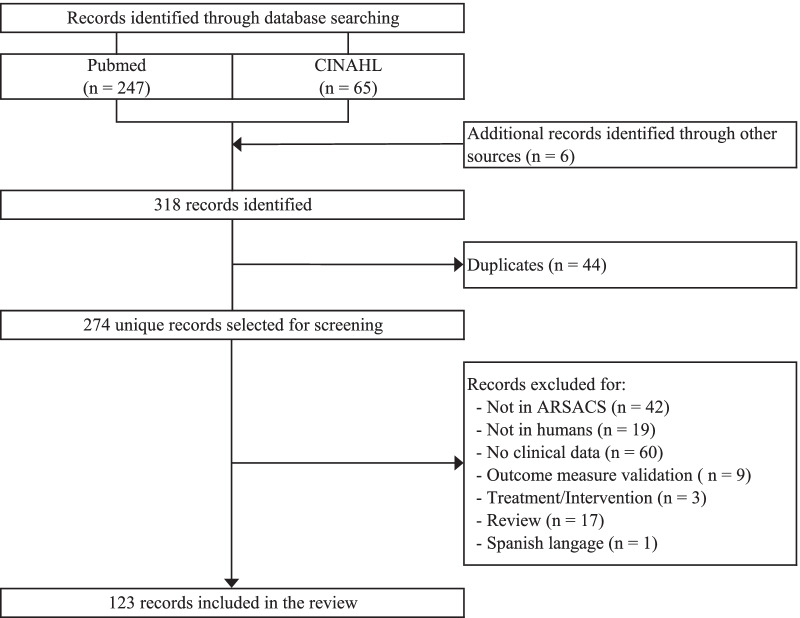
Flowchart of the systematic review of the literature

A systematic review of the literature was conducted to document the manifestations and impacts of ARSACS. The PubMed and CINHAL databases were consulted with the keywords “Spastic ataxia Charlevoix–Saguenay type", “ataxia of Charlevoix–Saguenay”, “Autosomal recessive spastic ataxia of Charlevoix–Saguenay”, “spastic ataxia of Charlevoix–Saguenay” and “Charlevoix–Saguenay spastic ataxia”. Additional records from other sources (secondary references) were included for a total of 318 articles. After removing duplicates, the title and abstract of all articles were reviewed, and those who met the exclusion criteria were excluded (Fig. 1). A total of 123 articles were included in the review. Data from the articles were extracted and classified in the blueprint following the domains of the PROMIS framework.

### Semistructured interviews

This part of the study took place at the Neuromuscular Clinic of the CIUSSS-SLSJ, where the greatest number of persons affected with ARSACS worldwide (n = 169) are followed. Inclusion criteria were to be 18 years old or older, to have a diagnosis of ARSACS confirmed by DNA testing and not to be affected by another condition causing significant functional limitations. Participants were selected by a purposive strategy ensuring diversity of their characteristics (sex, age and mobility level). Participants were recruited among clients of the Neuromuscular Clinic of the CIUSSS-SLSJ and were part of an international registry for recessive ataxia (n = 68) (PREPARE). Some strategies have been used to ensure appropriate saturation. First, the sample size was determined by a method developed by Francis et al. [45], where an initial analysis sample and a stopping criterion are decided a priori. Here, an initial sample of ten participants was recruited with a stopping criterion at two participants. Thus, ten interviews were realized and analyzed, and if there were no new emerging themes after two more interviews, it can be assumed that saturation was properly achieved. Therefore, the minimal sample size was 12 participants. To assess whether new themes emerged, a saturation table was used [68, 114], and the blueprint was used as a codebook [58]. An interview guide was developed using the conceptual framework PROMIS to ensure coverage of the entire participants’ experience. Open questions about their physical, mental, and social health and the impact of the disease on their daily life were asked, and participants completed a brief sociodemographic questionnaire. The interviews were conducted at the Neuromuscular Clinic or at the participant’s home (according to their preference) by a member of the research team with expertise in qualitative data collection methods (MT) and a patient research partner to ensure full understanding of the participants’ experience. The interviews were audio recorded and transcribed. NVivo 12 software was used to code the data with a half-opened coding strategy, and they were analyzed with the blueprint based on the PROMIS framework following a content analysis method [97].

### Discussion groups

Two discussion groups were realized with participants affected with a form of recessive ataxia other than ARSACS. The objective was to compare manifestations and impacts of the disease (differences and similarities) between those with ARSACS and those with another form of recessive ataxia. One of the groups took place at the Neuromuscular Clinic of the CIUSSS-SLSJ (group 1), and the other was realized at the Rehabilitation Center for Physical Disabilities of the *Centre intégré universitaire de santé et de services sociaux de la Capitale-Nationale* (Québec, Canada) (group 2). The participants were recruited by clinicians who used their clinical judgment to choose key informants. The inclusion criteria were to be 18 years old or older, to have a diagnosis of recessive ataxia other than ARSACS confirmed by DNA analysis and not to be affected by other conditions causing functional limitations. Similar to the interviews, the groups were conducted by the same two members. The groups were audio recorded, and the audiotapes were reviewed to conduct a content analysis [61]. The data gathered were added to the blueprint used in the analysis of the interviews in a distinct column to compare the experience of people with another recessive ataxia with the experience of people with ARSACS.

## Results part 1: comparison between the literature and interviews

A total of 12 persons with ARSACS participated in the semistructured interviews. Their characteristics can be seen in Table 1. The interviews took place between January 2019 and March 2019 and had a mean duration of approximately 45 min (app. 24–88 min).

**Table 1:** Characteristics of the study population for semistructured interviews (n = 12)

Characteristics	Total group	No walking difficulty (n = 3)	Walking aid (n = 4)	Wheelchair (n = 5)
Age				
Mean	39.3	23.7	44.3	44
Range	18–66	18–31	36–66	35–54
Sex (n)				
Female	6	1	3	2
Male	6	2	1	3

The results are reported following the PROMIS conceptual framework. Table 2 summarizes the results of the literature review and the semistructured interviews. It should be noted that in qualitative research using interviews, prevalence refers to the number of participants who mentioned the difficulty, which does not mean that the other participants do not experience this difficulty. This section presents only the difficulties that were self-reported by participants during the interviews in comparison with the literature. Refer to Table 3 for the other elements present in the literature that are not self-reported.

**Table 2:** Results of the review of literature and the semistructured interviews—self-reported findings

Subcomponent	Themes	Literature	Prevalence in the ARSACS sample (n = 12)
*Physical health*			
Symptoms	Pain	Bouchard et al. [13], Briand et al. [21], Çakar et al. [23], Dougherty et al. [36], Sahin et al. [116]	4
	Cramps and spasms	Bouchard et al. [13], Briand et al. [21], Gagnon et al. [46], Habibzadeh et al. [59], Leavitt et al. [74], Rezende Filho et al. [107]	5
	Fatigue	N/D^1^	10
Functions	Lower limbs and mobility		
	Impaired walking	Abkur et al. [1], Agarwal et al. [3], Agarwal et al. [2], Bourassa et al. [19], Breckpot et al. [20], Çakar et al. [23], Chen et al. [27], Cho et al. [28], Dougherty et al. [36], Grieco et al. [56], Gücüyener et al. [57], Habibzadeh et al. [59], Haga et al. [60], Kamada et al. [66], Kwon et al. [72], Lu et al. [79], McKenzie et al. [83], Martin et al. [80], Masciullo et al. [82], Miyatake et al. [87], Ogawa et al. [91], Pensabene et al. [102], Petrov [103], Prodi et al. [106], Rezende Filho et al. [107], Sahin et al. [116], Samanci et al. [117], Verhoeven et al. [135], Wagner et al. [140], Xiromerisiou et al. [143]	8
	Loss of mobility	Aida et al. [4], Bouchard [12], Bouchard et al. [14], Bouchard et al. [15], Cho et al. [28], El Euch-Fayache et al. [40], Gagnon et al. [46, 49], Gazulla et al. [52], Grieco et al. [56], Kwon et al. [72], Pensabene et al. [102], Petrov [103], Robitaille et al. [113], Sahin et al. [116], Samanci et al. [117], Sheetal et al. [119], Shimazaki et al. [120], Srikajon et al. [123], Terracciano et al. [128], Tzoulis et al. [131]	N/D
	Use of walking aid	Aida et al. [4], Al-Ajmi et al. [5], Anheim et al. [7], Bourassa et al. [19], Breckpot et al. [20], Gagnon et al. [48], Garcia et al. [51], Haga et al. [60], Hara et al. [63], Incecik et al. [64], Kamada et al. [66], Leavitt et al. [74], Lessard et al. [76], Liew et al. [77], Miyatake et al. [87], Narayanan et al. [89], Ogawa et al. [91], Palmio et al. [99], Richter et al. [112], Sahin et al. [116], Samanci et al. [117], Shimazaki et al. [120], Srikajon et al. [123], Terracciano et al. [128], van Lint et al. [133], Verhoeven et al. [134], Wang et al. [141]	N/D
	Stiffness	Agarwal et al. [2], Bouchard et al. [13], Breckpot et al. [20], Griecio (2004), Haga et al. [60], Leavitt et al. [74], Samanci et al. [117], Wagner et al. [140]	7
	Foot abnormalities	Abkur et al. [1], Agarwal et al. [2], Agarwal et al. [3], Al-Ajmi et al. [5], Ali et al. [6], Anheim et al. [7], Baets et al. [9], Bouchard [12], Bouchard et al. [13], Burguêz et al. [22], Çakar et al. [23], Chen et al. [27], Criscuolo et al. [30], Desserre et al. [34], Duquette et al. [38], Dziewulska [39], El Euch-Fayache et al. [40], Gazulla et al. [52], Gazulla et al. [53], Gazulla et al. [54], Gregianin et al. [55], Grieco et al. [56], Habibzadeh et al. [59], Hamza et al. [62], Hara et al. [63], Incecik et al. [64], Kamada et al. [66], Krygier et al. [69], Kwon et al. [72], Leavitt et al. [74], Liu et al. [78], Lu et al. [79], Masciullo et al. [81], McKenzie et al. [83], Mignarri et al. [85], Miyatake et al. [87], Ogawa et al. [91], Okawa et al. [93], Ouyang et al. [95], Pablo et al. [96], Palmio et al. [99], Parkinson et al. [100], Pedroso et al. [101], Pensabene et al. [102], Prodi et al. [106], Ricca et al. [110], Rezende Filho et al. [107], Ricca et al. [109], Saffie et al. [115], Sahin et al. [116], Shimazaki et al. [120], Shimazaki et al. [122], Terracciano et al. [128], Terracciano et al. [129], Vermeer et al. [136], Vill et al. [137], Wang et al. [141]	2
	Balance	Abkur et al. [1], Agarwal et al. [2], Agarwal et al. [3], Al-Ajmi et al. [5], Bouchard [12], Bouchard et al. [13], Bouchard and Langlois [16], Bourassa et al. [19], Burguêz et al. [22], Cho et al. [28], Dougherty et al. [36], Gagnon et al. [46], Habibzadeh et al. [59], Incecik et al. [64], Kuchay et al. [71], Liu et al. [78], Lu et al. [79], McKenzie et al. [83], Miyatake et al. [87], Narayanan et al. [89], Palmio et al. [99], Prodi et al. [106], Sahin et al. [116], Samanci et al. [117], Sheetal et al. [119], Srikajon et al. [123], van Lint et al. [133], Wagner et al. [140], Wang et al. [141], Xiromerisiou et al. [143], Yamamoto et al. [144]	11
	Upper limbs		
	Dexterity	Gagnon et al. [47], Incecik et al. [64], Martin et al. [80], Okawa et al. [93], Sahin et al. [116], Sheetal et al. [119], Terracciano et al. [129], Wang et al. [141]	9
	Strength	Bouchard et al. [13, 14], Bourassa et al. [19], Gazulla et al. [52], Gregianin et al. [55], Grieco et al. [56], Hara et al. [63], Kamada et al. [66], Leavitt et al. [74], Okawa et al. [93], Palmio et al. [99], Parkinson et al. [100], Picher-Martel and Dupre [104], Ricca et al. [109], Richards et al. [111], Vermeer et al. [136], Wang et al. [141]	N/D
	Muscle atrophy and weakness	Abkur et al. [1], Aida et al. [4], Al-Ajmi et al. [5], Ali et al. [6], Anheim et al. [7], Baets et al. [9], Bouchard et al. [13], Breckpot et al. [20], Burguêz et al. [22], Çakar et al. [23], Chen et al. [27], Dougherty et al. [36], Gagnon et al. [47], Gazulla et al. [52], Gazulla et al. [54], Gregianin et al. [55], Grieco et al. [56], Hara et al. [63], Kamada et al. [66], Krygier et al. [69], Kuchay et al. [71], Leavitt et al. [74], Liu et al. [78], Lu et al. [79], Masciullo et al. [82], McKenzie et al. [83], Miyatake et al. [87], Okawa et al. [93], Palmio et al. [99], Parkinson et al. [100], Picher-Martel and Dupre [104], Pensabene et al. [102], Prodi et al. [106], Ricca et al. [109], Ricca et al. [110], Richards et al. [111], Samanci et al. [117], Shimazaki et al. [121], Shimazaki et al. [122], Terracciano et al. [129], Tzoulis et al. [131], Vermeer et al. [136], Vill et al. [137], Xiromerisiou et al. [143], Yamamoto et al. [144]	8
	Coordination	Gagnon et al. [47], Leavitt et al. [74], Lessard et al. [76], Lu et al. [79], McKenzie et al. [83], Oguz et al. [92], Sheetal et al. [119]	6
	Bladder and bowel functions		4
	Urinary urgency	Bouchard et al. [13], El Euch-Fayache et al. [40], Gregianin et al. [55], Grieco et al. [56], Leavitt et al. [74], McKenzie et al. [83], Mignarri et al. [85], Prodi et al. [106], Synofzik et al. [127], Terracciano et al. [129], Tzoulis et al. [131], Vermeer et al. [136], Wang et al. [141]	N/D
	Urinary incontinence	Bouchard [12], Bouchard et al. [13], Gregianin et al. [55], Mignarri et al. [85], Miyatake et al. [87], Sahin et al. [116], Synofzik et al. [127], Tzoulis et al. [131]	N/D
	Fecal urgency	Bouchard [12], Briand et al. [21], Gregianin et al. [55], Synofzik et al. [127]	N/D
	Other bladder and bowel dysfunctions	Dziewulska [39], Miyatake et al. [87], Palmio et al. [99], Rezende Filho et al. [107], Yamamoto et al. [144]	N/D
	Sexual functions	Miyatake et al. [87], Synofzik et al. [127]	2
	Dysarthria	Agarwal et al. [2], Agarwal et al. [3], Aida et al. [4], Al-Ajmi et al. [5], Ali et al. [6], Anheim et al. [7], Baets et al. [9], Blumkin et al. [10], Borruat et al. [11], Bouchard [12], Bouchard et al. [13], Bouchard et al. [15], Bouchard and Langlois [16], Bouhlal et al. [18], Breckpot et al. [20], Burguêz et al. [22], Chen et al. [27], Cho et al. [28], Criscuolo et al. [30], Desserre et al. [34], Duquette et al. [38], Dziewulska [39], El Euch-Fayache et al. [40], Garcia et al. [51], Gazulla et al. [53], Grieco et al. [56], Gücüyener et al. [57], Habibzadeh et al. [59], Haga et al. [60], Hamza et al. [62], Hara et al. [63], Incecik et al. [64], Kamada et al. [66], Karuvath et al. [67], Krygier et al. [69], Kuchay et al. [71], Kwon et al. [72], Leavitt et al. [74], Lu et al. [79], Martin et al. [80], Masciullo et al. [82], McKenzie et al. [83], Mignarri et al. [85], Miyatake et al. [87], Narayanan et al. [89], Ogawa et al. [91], Oguz et al. [92], Ouyang et al. [95], Palmio et al. [99], Pedroso et al. [101], Pensabene et al. [102], Petrov [103], Ricca et al. [110], Richter et al. [112], Robitaille et al. [113], Sahin et al. [116], Samanci et al. [117], Sheetal et al. [119], Shimazaki et al. [120], Shimazaki et al. [122], Srikajon et al. [123], Stevens et al. [124], Synofzik et al. [127], Terracciano et al. [128], Terracciano et al. [129], Tzoulis et al. [131], Van Damme et al. [132], van Lint et al. [133], Verhoeven et al. [134], Vermeer et al. [136], Vill et al. [137], Vingolo et al. [138], Vogel et al. [139], Wagner et al. [140], Wang et al. [141], Yamamoto et al. [144]	5
	Eye function	Abkur et al. [1], Agarwal et al. [2], Agarwal et al. [3], Aida et al. [4], Al-Ajmi et al. [5], Anheim et al. [7], Anheim et al. [8], Baets et al. [9], Blumkin et al. [10], Borruat et al. [11], Bouchard [12], Bouchard et al. [13], Bouchard et al. [15], Bouchard and Langlois [16], Bouhlal et al. [18], Burguêz et al. [22], Çakar et al. [23], Cho et al. [28], Criscuolo et al. [30], Desserre et al. [34], Dionne et al. [35], Dougherty et al. [36], Douglas et al. [37], Duquette et al. [38], Dziewulska [39], El Euch-Fayache et al. [40], Garcia et al. [51], Gazulla et al. [52], Gazulla et al. [53], Gazulla et al. [54], Grieco et al. [56], Gücüyener et al. [57], Habibzadeh et al. [59], Hamza et al. [62], Hara et al. [63], Incecik et al. [64], Kamada et al. [66], Krygier et al. [69], Kuchay et al. [71], Kwon et al. [72], Leavitt et al. [74], Liew et al. [77], Liu et al. [78], Lu et al. [79], Masciullo et al. [81], McKenzie et al. [83], McMillan et al. [84], Miyatake et al. [87], Narayanan et al. [89], Ogawa et al. [91], Oguz et al. [92], Okawa et al. [93], Ouyang et al. [95], Pablo et al. [96], Palmio et al. [99], Parkinson et al. [100], Pedroso et al. [101], Pensabene et al. [102], Picher-Martel and Dupre [104], Rezende Filho et al. [107], Rezende Filho et al. [108], Ricca et al. [109], Richter et al. [112], Sahin et al. [116], Samanci et al. [117], Sheetal et al. [119], Shimazaki et al. [120], Shimazaki et al. [121], Stevens et al. [124], Srikajon et al. [123], Synofzik et al. [127], Terracciano et al. [128], Terracciano et al. [129], Tzoulis et al. [131], van Lint et al. [133], Vermeer et al. [136], Vill et al. [137], Vingolo et al. [138], Wang et al. [141], Xiromerisiou et al. [143], Yamamoto et al. [144], Yu-Wai-Man et al. [145]	2
	Dysphagia	Bouchard et al. [13], Cho et al. [28], Gagnon et al. [48], Grieco et al. [56], McKenzie et al. [83], Miyatake et al. [87], Prodi et al. [106], Rezende Filho et al. [107], Sahin et al. [116], Samanci et al. [117], Shimazaki et al. [120], Terracciano et al. [129], Tzoulis et al. [131], Vermeer et al. [136], Vingolo et al. [138], Vogel et al. [139]	5
	Physical activities	Aida et al. [4], Dougherty et al. [36], Habibzadeh et al. [59], Haga et al. [60], Ouyang et al. [94], Sheetal et al. [119], Shimazaki et al. [120], Wagner et al. [140], Wang et al. [141], Xiromerisiou et al. [143], Yamamoto et al. [144]	5
	ADLs	Bourassa et al. [19], Gagnon et al. [47], Gagnon et al. [48], Petrov [103]	7
*Mental health*			
Affect	Anxiety	Forgue et al. [44], Mignarri et al. [85]	2
	Depression	Forgue et al. [44], Mignarri et al. [85], Petrov [103]	4
	Frustration	Mignarri et al. [85]	6
	Negative psychosocial impact of illness	Forgue et al. [44]	2
Cognition	Cognitive abilities	Ali et al. [6], Bouchard et al. [13, 14], Breckpot et al. [20], Çakar et al. [23], Dougherty et al. [36], Gücüyener et al. [57], Kamada et al. [66], Kuchay et al. [71], Mignarri et al. [85], Ogawa et al. [91], Oguz et al. [92], Okawa et al. [93], Petrov [103], Pilliod et al. [105], Prodi et al. [106], Ricca et al. [109], Richter et al. [112], Shimazaki et al. [121], Terracciano et al. [128], Verhoeven et al. [134], Yamamoto et al. [144]	10
	Memory	Briand et al. [21], Hara et al. [63], Krygier et al. [69]	N/D
	Attention	Briand et al. [21]	N/D
	Other cognitive dysfunctions	Desserre et al. [34], Duquette et al. [38], Hamza et al. [62], Hara et al. [63], Lu et al. [79], Prodi et al. [106], Tzoulis et al. [131]	N/D
*Social health*			
Relationships		Forgue et al. [44]	6
Function	Work	Bourassa et al. [19], Forgue et al. [44], Tremblay et al. [130], Verhoeven et al. [134]	12
	Studies	Breckpot et al. [20], Duquette et al. [38], Grieco et al. [56], Kamada et al. [66], Pilliod et al. [105], Prodi et al. [106], Sheetal et al. [119]	5
	Parenthood	N/D	2
	Social activities	Forgue et al. [44], Gagnon et al. [46], Gagnon et al. [47], Gagnon et al. [49]	9

^1^Not documented

**Table 3:** Other results of the review of the literature

Components	Subcomponents	Literature
Audition		Briand et al. [21]
Nervous system		
	Epilepsy	Çakar et al. [23], Duquette et al. [38], Wang et al. [141]
	Dizziness	Bouchard et al. [13]
Locomotor functions	Tremor	Abkur et al. [1], Bouchard et al. [13], Dougherty et al. [36], Incecik et al. [64], Liew et al. [77], McKenzie et al. [83], Narayanan et al. [89], Palmio et al. [99], Pensabene et al. [102], Sahin et al. [116], Samanci et al. [117], Sheetal et al. [119], Vill et al. [137]
	Brisk/hyperreflexia	Agarwal et al. [2], Anheim et al. [7], Blumkin et al. [10], Borruat et al. [11], Bouchard [12], Bouchard et al. [13], Bouchard et al. [15], Breckpot et al. [20], Burguêz et al. [22], Criscuolo et al. [30], Dougherty et al. [36], Duquette et al. [38], Dziewulska [39], El Euch-Fayache et al. [40], Garcia et al. [51], Gregianin et al. [55], Grieco et al. [56], Habibzadeh et al. [59], Haga et al. [60], Hamza et al. [62], Hara et al. [63], Incecik et al. [64], Karuvath et al. [67], Liu et al. [78], Martin et al. [80], McKenzie et al. [83], Ogawa et al. [91], Okawa et al. [93], Parkinson et al. [100], Pedroso et al. [101], Picher-Martel and Dupre [104], Prodi et al. [106], Rezende Filho et al. [107], Richter et al. [112], Saffie et al. [115], Samanci et al. [117], Sheetal et al. [119], Shimazaki et al. [120], Shimazaki et al. [121], Terracciano et al. [129], Vingolo et al. [138], Wagner et al. [140], Xiromerisiou et al. [143], Yamamoto et al. [144]
	Tendon reflex absent/decreased	Agarwal et al. [3], Aida et al. [4], Al-Ajmi et al. [5], Anheim et al. [7], Bouchard [12], Bouchard et al. [13], Bouchard et al. [15], Bouhlal et al. [18], Breckpot et al. [20], Burguêz et al. [22], Chen et al. [27], Criscuolo et al. [30], Desserre et al. [34], Dougherty et al. [36], Duquette et al. [38], El Euch-Fayache et al. [40], Gazulla et al. [52], Grieco et al. [56], Gücüyener et al. [57], Habibzadeh et al. [59], Hara et al. [63], Kamada et al. [66], Krygier et al. [69], Kuchay et al. [71], Kwon et al. [72], Leavitt et al. [74], Liu et al. [78], Mignarri et al. [85], Miyatake et al. [87], Narayanan et al. [89], Ogawa et al. [91], Okawa et al. [93], Palmio et al. [99], Parkinson et al. [100], Prodi et al. [106], Sahin et al. [116], Shimazaki et al. [120], Shimazaki et al. [121], Shimazaki et al. [122], Terracciano et al. [128], Terracciano et al. [129], Verhoeven et al. [134], Vermeer et al. [136], Vill et al. [137], Wang et al. [141], Xiromerisiou et al. [143], Yamamoto et al. [144]
	Clonus	Gregianin et al. [55], Hara et al. [63], Incecik et al. [64], Karuvath et al. [67], Liu et al. [78], Martin et al. [80], Narayanan et al. [89], Prodi et al. [106]
	Dystonia	Gazulla et al. [52], Lu et al. [79], McKenzie et al. [83], Oguz et al. [92], Rezende Filho et al. [107], Vermeer et al. [136]
	Dysmetria	Agarwal et al. [2], Agarwal et al. [3], Baets et al. [9], Borruat et al. [11], Chen et al. [27], Criscuolo et al. [30], Dougherty et al. [36], Gazulla et al. [52], Gazulla et al. [53], Grieco et al. [56], Martin et al. [80], Masciullo et al. [82], Pensabene et al. [102], Ricca et al. [110], Ricca et al. [109], Srikajon et al. [123], Synofzik et al. [127], Terracciano et al. [128], Vill et al. [137], Xiromerisiou et al. [143]
	Spasticity	Agarwal et al. [2], Agarwal et al. [3], Anheim et al. [7], Borruat et al. [11], Bouchard [12], Bouchard et al. [13], Bouchard et al. [15], Breckpot et al. [20], Burguêz et al. [22], Çakar et al. [23], Criscuolo et al. [30], Desserre et al. [34], Dougherty et al. [36], Douglas et al. [37], Duquette et al. [38], Dziewulska [39], El Euch-Fayache et al. [40], Gazulla et al. [52], Gazulla et al. [53], Gazulla et al. [54], Gregianin et al. [55], Grieco et al. [56], Gücüyener et al. [57], Haga et al. [60], Hamza et al. [62], Hara et al. [63], Incecik et al. [64], Krygier et al. [69], Kuchay et al. [71], Kwon et al. [72], Leavitt et al. [74], Lessard et al. [76], Liew et al. [77], Martin et al. [80], Masciullo et al. [81], Narayanan et al. [89], Ogawa et al. [91], Oguz et al. [92], Okawa et al. [93], Pablo et al. [96], Palmio et al. [99], Parkinson et al. [100], Pedroso et al. [101], Pensabene et al. [102], Petrov [103], Pilliod et al. [105], Rezende Filho et al. [107], Richter et al. [112], Sahin et al. [116], Samanci et al. [117], Sheetal et al. [119], Shimazaki et al. [120], Shimazaki et al. [121], Srikajon et al. [123], Synofzik et al. [127], Terracciano et al. [129], Tzoulis et al. [131], Vermeer et al. [136], Yamamoto et al. [144]
	Ataxia	Agarwal et al. [2], Agarwal et al. [3], Aida et al. [4], Al-Ajmi et al. [5], Ali et al. [6], Anheim et al. [7], Baets et al. [9], Blumkin et al. [10], Borruat et al. [11], Bouchard [12], Bouchard et al. [13], Bouchard et al. [15], Bouhlal et al. [18], Breckpot et al. [20], Burguêz et al. [22], Çakar et al. [23], Criscuolo et al. [30], Duquette et al. [38], Gagnon et al. [47], Garcia et al. [51], Gazulla et al. [52], Gazulla et al. [54], Grieco et al. [56], Habibzadeh et al. [59], Hamza et al. [62], Incecik et al. [64], Kamada et al. [66], Krygier et al. [69], Kuchay et al. [71], Kwon et al. [72], Leavitt et al. [74], Liu et al. [78], Lu et al. [79], Martin et al. [80], Masciullo et al. [81], Masciullo et al. [82], McKenzie et al. [83], Mignarri et al. [85], Miyatake et al. [87], Narayanan et al. [89], Ogawa et al. [91], Oguz et al. [92], Okawa et al. [93], Ouyang et al. [95], Pablo et al. [96], Palmio et al. [99], Parkinson et al. [100], Pedroso et al. [101], Picher-Martel and Dupre [104], Pilliod et al. [105], Rezende Filho et al. [107], Ricca et al. [110], Ricca et al. [109], Saffie et al. [115], Sahin et al. [116], Samanci et al. [117], Shimazaki et al. [120], Shimazaki et al. [121], Shimazaki et al. [122], Srikajon et al. [123], Terracciano et al. [128], Terracciano et al. [129], Tzoulis et al. [131], Verhoeven et al. [134], Vermeer et al. [136], Vill et al. [137], Yamamoto et al. [144], Xiromerisiou et al. [143]
	Distal atrophy	Ali et al. [6], Anheim et al. [7], Bouchard [12], Bouchard et al. [13, 14], Breckpot et al. [20], Criscuolo et al. [30], Desserre et al. [34], Duquette et al. [38], Dziewulska [39], El Euch-Fayache et al. [40], Gagnon et al. [49], Garcia et al. [51], Gazulla et al. [53], Gregianin et al. [55], Grieco et al. [56], Haga et al. [60], Hara et al. [63], Kamada et al. [66], Krygier et al. [69], Kuchay et al. [71], Leavitt et al. [74], Liu et al. [78], Martin et al. [80], Masciullo et al. [81], McKenzie et al. [83], Miyatake et al. [87], Okawa et al. [93], Ouyang et al. [95], Palmio et al. [99], Pedroso et al. [101], Prodi et al. [106], Rezende Filho et al. [107], Robitaille et al. [113], Shimazaki et al. [120], Terracciano et al. [128], Tzoulis et al. [131], Verhoeven et al. [134], Vermeer et al. [136], Vill et al. [137], Vingolo et al. [138]
	Babinski sign	Anheim et al. [7], Bouchard [12], Bouchard et al. [13], Bouchard et al. [15], Burguêz et al. [22], Chen et al. [27], Criscuolo et al. [30], Duquette et al. [38], Garcia et al. [51], Gazulla et al. [52], Gazulla et al. [53], Gregianin et al. [55], Grieco et al. [56], Hamza et al. [62], Hara et al. [63], Incecik et al. [64], Kamada et al. [66], Kuchay et al. [71], Kwon et al. [72], Liu et al. [78], Martin et al. [80], Mignarri et al. [85], Miyatake et al. [87], Narayanan et al. [89], Ogawa et al. [91], Okawa et al. [93], Ouyang et al. [95], Palmio et al. [99], Pensabene et al. [102], Petrov [103], Prodi et al. [106], Rezende Filho et al. [107], Ricca et al. [110], Ricca et al. [109], Richter et al. [112], Robitaille et al. [113], Sahin et al. [116], Samanci et al. [117], Shimazaki et al. [120], Shimazaki et al. [121], Shimazaki et al. [122], Srikajon et al. [123], Synofzik et al. [127], Terracciano et al. [128], Terracciano et al. [129], Verhoeven et al. [134], Vingolo et al. [138], Wagner et al. [140], Wang et al. [141], Xiromerisiou et al. [143], Yamamoto et al. [144]
	Fasciculations	Leavitt et al. [74]
	Pallesthesia of the lower limbs	Anheim et al. [7], Gazulla et al. [52], Gregianin et al. [55], Kamada et al. [66], McKenzie et al. [83], Ogawa et al. [91], Palmio et al. [99], Rezende Filho et al. [107], Shimazaki et al. [120], Vill et al. [137], Vingolo et al. [138], Yamamoto et al. [144]
	Posture	Bouhlal et al. [17], Dougherty et al. [36], Gazulla et al. [52], Gazulla et al. [53], Gregianin et al. [55], Tzoulis et al. [131], Vingolo et al. [138]
	Postural control	Shimazaki et al. [120]
	Paroxysmal *kinesigenic choreoathetosis*	Briand et al. [21]
	*Dysdiachokinesia*	Agarwal et al. [2]
	*Rigidity*	Habibzadeh et al. [59]
	*Mirror movements*	Habibzadeh et al. [59]
	*Hypokinesia*	Habibzadeh et al. [59]
	Bradikinesia	Wagner et al. [140]
Childhood development	Psychomotor delays	Agarwal et al. [3], Anheim et al. [7], Bouchard et al. [15], Dougherty et al. [36], Gregianin et al. [55], Grieco et al. [56], Mignarri et al. [85], Oguz et al. [92]
Sensory system	Peripheral neuropathy of the lower limbs	Abkur et al. [1], Briand et al. [21], Dziewulska [39], Kuchay et al. [71], Pedroso et al. [101], Samanci et al. [117], Tzoulis et al. [131]
	Peripheral neuropathy	Al-Ajmi et al. [5], Ali et al. [6], Agarwal et al. [3], Anheim et al. [8], Bouhlal et al. [18], Breckpot et al. [20], Desserre et al., Dougherty et al., Duquette et al. [38], Gregianin et al. [55], Grieco et al. [56], Krygier et al. [69], Krygier et al. [70], Liew et al. [77], Martin et al. [80], Masciullo et al. [81], Miyatake et al. [87], Oguz et al. [92], Palmio et al. [99], Pedroso et al. [101], Pensabene et al. [102], Pilliod et al. [105], Rezende Filho et al. [107], Ricca et al. [109], Saffie et al. [115], Shimazaki et al. [121], Synofzik et al. [127], Terracciano et al. [129], Verhoeven et al. [134], Vermeer et al. [136], Yu-Wai-Man et al. [145]
	Neuropathy	Al-Ajmi et al. [5], Baets et al. [9], Bouchard [12], Bouchard et al. [13], Bouhlal et al. [18], Çakar et al. [23], Chen et al. [27], Cho et al. [28], El Euch-Fayache et al. [40], Gazulla et al. [53], Gazulla et al. [54], Gücüyener et al. [57], Hara et al. [63], Kamada et al. [66], Karuvath et al. [67], Krygier et al. [69], Kwon et al. [72], Leavitt et al. [74], Liu et al. [78], Mignarri et al. [85], Miyatake et al. [87], Okawa et al. [93], Parkinson et al. [100], Pedroso et al. [101], Ricca et al. [110], Rezende Filho et al. [107], Sahin et al. [116], Sheetal et al. [119], Shimazaki et al. [122], Srikajon et al. [123], Stevens et al. [124], Tzoulis et al. [131], Verhoeven et al. [134], Vermeer et al. [136], Vill et al. [137], Wang et al. [141], Xiromerisiou et al. [143], Yamamoto et al. [144]
	Paresis distal	Breckpot et al. [20], Dziewulska [39], Gregianin et al. [55], Terracciano et al. [128], van Lint et al. [133], Verhoeven et al. [134], Vill et al. [137]
	Paresthesis	Leavitt et al. [74], Sahin et al. [116]
	Proprioception	Kamada et al. [66], Sahin et al. [116], Samanci et al. [117], Sheetal et al. [119], Srikajon et al. [123]
Musculoskeletal deformations	Distal	Bouchard and Langlois [16]
	Upper limbs	Bouchard and Langlois [16]
	Claw hand	Ali et al. [6], Bouchard et al. [13], Dougherty et al. [36], Ogawa et al. [91], Okawa et al. [93], Shimazaki et al. [120], Shimazaki et al. [122]
	Scoliosis	Çakar et al. [23], Criscuolo et al. [30], Desserre et al. [34], El Euch-Fayache et al. [40], Hamza et al. [62], Prodi et al. [106]
	Bone density	McKenzie et al. [83]

## Physical health

The first component of the PROMIS conceptual framework is physical health. In this component, there are symptoms and functions as subcomponents. Pain and fatigue are the two symptoms identified in the review of the literature.

### Pain

Little is known in the literature about pain in ARSACS. about the authors discuss neuropathic pain in the lower limbs [21, 23], pain in the lower limbs [116], headache [36] and painful cramps in the neck, upper limbs and lower limbs [13]. Spasms and cramps have also been reported by other authors [21, 46, 48, 49, 59, 74, 107]. The results show that 4/12 participants felt pain in different body parts (knees, shoulders, back, neck, hands, wrists, ankles, legs). Otherwise, 5/12 participants reported muscle cramps or spasms (upper and lower limbs) that could be painful. These symptoms can limit activities such as using a computer, performing transfers or hindering sleep.

### Fatigue

Fatigue is a symptom in ARSACS that is not documented in the literature. However, 10/12 participants reported that they experienced fatigue as they had trouble managing their energy or had little endurance. Energy management is reported as a lack of energy that has many impacts on the accomplishment of activities of daily living (ADLs), as the people affected have to implement several coping strategies to deal with fatigue (e.g., prioritize tasks, take naps, use technical or human assistance). The lack of endurance (perform a task for a long time) makes affected people feel exhausted rapidly and, like energy management, affects daily life and requires adaptations. Experience fatigue has consequences on performing daily activities and social roles (work, study) and can increase the severity of other manifestations, such as balance.

The other subcomponent of physical health is functions. There are several functions that are affected by ARSACS: lower limbs, upper limbs, balance, strength, coordination, bladder and bowel functions, sexual functions, dysarthria, eye function, dysphagia, physical activities, and ADLs.

### Lower limbs and mobility

Even if the literature in ARSACS is scarce, several papers report impaired walking [1–3, 19, 20, 23, 27, 28, 36, 56, 57, 59, 60, 66, 72, 79–81, 83, 87, 91, 102, 103, 106, 107, 116, 117, 135, 140, 143], progressive loss of mobility [4, 12, 14, 15, 28, 40, 46, 48, 49, 52, 56, 72, 76, 102, 103, 113, 116, 117, 119, 120, 123, 128, 131] and the need to use a walking aid early on and progressively move on to a wheelchair [4, 5, 7, 19, 20, 46, 48, 49, 51, 60, 63, 64, 66, 74, 76, 77, 87, 89, 91, 99, 112, 116, 117, 120, 123, 128, 133, 134, 141]. In the sample, 8/12 participants reported the impacts of this progressive loss of mobility. This includes difficulty or incapacity to walk, which may involve the use of a walking aid, inactivity and weight gain, financial impacts related to purchasing technical aids and adaptations and a decrease in independence.

Stiffness is also documented in the literature [2, 13, 20, 56, 60, 117, 131, 140]. Qualitative interviews revealed that 7/12 participants experienced stiffness that made it difficult for them to be flexible and perform certain tasks, such as putting on shoes or getting into a bath, in addition to an increased risk of falling related to stumbling. They describe it as having “heavy legs” that make them less agile.

Some foot deformities are also reported in ARSACS, such as pes cavus, hammer toes and foot drop, without clear prevalence [1–3, 5–7, 9, 12, 13, 22, 23, 27, 30, 34, 38–40, 52, 55, 56, 59, 62–64, 66, 69, 72, 74, 78, 79, 81, 91, 93, 95, 96, 99–102, 106, 107, 109, 110, 115, 116, 120, 122, 129, 136, 137, 141]. Only 2/12 participants reported foot deformities that impacted their life. They observed a pronounced arch of the feet that can be painful and needs orthoses.

### Balance

Balance difficulties are clearly reported in the literature [1–3, 5, 12, 13, 19, 22, 28, 36, 46, 48, 49, 59, 64, 71, 76, 78–80, 83, 87, 89, 99, 106, 116, 117, 119, 123, 133, 140, 141, 143, 144]. Almost all participants reported experiencing problems with balance. There are three major issues identified with loss of balance: risk of falling, limitation with ADLs, and limitation with physical activities. Loss of balance impairs gait and increases the risk of falling. These falls can cause injuries, and they often need to hold on something or to aid to avoid it. Performing ADLs can also be compromised by the loss of balance. They often need to hold on something (e.g., wall, counter, grab bar) when doing their everyday activities such as cooking, bathing, or doing household chores. Transporting objects with liquid in them (e.g., glass of water, soup) is challenging for many of them. The loss of balance can also impair the ability to perform physical activities such as team sports and winter activities, as some people had to quite performing sports they like because of it. Some participants talked about the social impact of loss of balance as people they encountered thought they were intoxicated by alcohol.

### Upper limbs

For the upper limbs, the literature suggests difficulties with fine movement, clumsiness, and variable difficulties with dexterity [47, 64, 80, 93, 116, 119, 129, 141]. Otherwise, a majority of participants (9/12) reported troubles with manual dexterity. These difficulties lead to major problems in everyday life, as they take more time and energy to perform simple tasks (e.g., cooking, button, drive) and can be very frustrating. Participants also reported trouble writing that can cause difficulties in schooling and for some work-related tasks.

### Strength

Even if strength seems normal for affected children, progressive and variable disabilities in strength are seen in ARSACS [19, 20, 47, 111, 141], in addition to muscle atrophy and weakness that affect both upper and lower limbs [1, 4–7, 9, 13, 20, 22, 23, 27, 36, 46–49, 52, 54–56, 63, 66, 69, 71, 74, 78, 79, 82, 83, 87, 93, 99, 100, 102, 104, 106, 109–111, 117, 121, 122, 129, 131, 136, 137, 143, 144]. Data showed that 8/12 participants reported muscle weakness, particularly in the legs, ankles, and hands. This weakness limits some daily activities, such as bending and getting up, lifting objects, cooking and performing physical activities. Some people (4/12) also said that muscle weakness increases the risk of falling and the capacity to raise after a fall.

### Coordination

Some authors suggest variable difficulties with coordination [47, 74, 76, 79, 83, 92, 119]. Half of the participants of the study talked about the impacts of coordination difficulties in terms of limitations in their ADL and physical activities. The participants reported difficulty in performing simultaneous tasks, such as when, cooking, driving or doing sports.

### Bladder and bowel functions

In the literature, there is much involvement of bladder and bowel functions in ARSACS. The authors reported urinary urgency [13, 40, 55, 56, 74, 83, 85, 106, 127, 129, 131, 136, 141], urinary incontinence [12, 13, 55, 85, 87, 116, 127, 131], fecal urgency [12, 21, 55, 127] and other bladder and bowel dysfunctions, such as constipation and diarrhea [39, 87, 99, 107, 144]. Nearly half of the participants (4/12) reported difficulty retaining urine or stool and that these difficulties can limit their social activities (e.g., long trip, swimming). They mostly talk about urgency (urinary and fecal) that can lead to incontinence.

### Sexual functions

As expected, sexual functioning is rarely addressed in the literature. Only two articles discussed erectile dysfunction [87, 127]. At the same time, only two participants (identified as female) talked about their sexual functioning, as they noted a lack of flexibility that made some sexual positions harder to achieve and pain (postpartum).

### Dysarthia

A wide range of dysarthria severity (mild to severe) are documented [2–7, 9–13, 15, 16, 18, 20, 22, 27, 28, 30, 34, 38–40, 51, 53, 56, 57, 59, 60, 62–64, 66, 67, 69, 71, 72, 74, 79, 80, 82, 83, 85, 87, 89, 91, 92, 95, 99, 101–103, 110, 112, 113, 116, 117, 119, 120, 122–124, 127–129, 131–134, 136–141, 144]. The authors described dysarthria in terms of slurred or unclear speech [3, 22, 66, 87, 103, 134], delay in speech production [140], and slow speech [141, 144]. Among the participants, 5/12 noted difficulty pronouncing words or slow speech. This makes it difficult for them to participate in an animated discussion or to express themselves. They often have to repeat, and people they meet tend to realize they have a disease by their distinctive speech.

### Eye functions

People with ARSACS can experience nystagmus, impairment in visuomotor coordination, abnormality in eye pursuit, decrease in visual acuity, and other ocular problems [1–5, 7–13, 15, 16, 18, 22, 23, 28, 30, 34–40, 51–54, 56, 57, 59, 62–64, 66, 69, 71, 72, 74, 77–79, 81, 83, 84, 87, 89, 91–93, 95, 96, 99–102, 104, 107–109, 112, 116, 117, 119, 120, 123, 124, 127–129, 131, 133, 136–138, 141, 143–145]. However, the participants in the study mostly did not perceive disturbances in eye functioning. Only two participants noted a decrease in visual acuity, which is not thought to be related to ARSACS.

### Dysphagia

A variable range of swallowing difficulties is reported in the literature. These difficulties can be related to dysphagia for liquids only [56, 83, 129], for both liquids and solids [13, 46, 48, 49, 139] or unspecified [28, 87, 106, 107, 116, 117, 120, 131, 136, 138]. According to Vogel et al. [139], dysphagia is more related to swallowing timing than it is to weakness, and it can lead to changes in eating and drinking habits. For the participants, 5/12 reported difficulties swallowing liquids, solids, both or their own saliva that can lead to choking. Few patients reported needing assistance during meal times or while drinking, however, 4/11 modified their eating and drinking habits to improve their swallowing (e.g., avoiding difficult-to-swallow foods).

### Physical activities

For people with ARSACS, there is a progressive loss of mobility that leads to difficulty or incapacity to run that begins in childhood [36, 59, 60, 94, 119, 120, 141, 144]. Thus, it creates difficulties in performing sports, such as team sports or gymnastics [4, 94, 140, 141, 143]. As seen before, people in the study reported difficulties in performing physical activities in relation to their physical impairment, such as incoordination and loss of balance. However, sometimes, they cannot identify the specific physical disability that hinders difficulty in performing physical activities, or it is a set of factors that make the cause difficult to identify. Among the participants, 5/12 had trouble performing physical activities. Thus, they had to choose activities adapted to their capacities, and it is sometime bringing dissatisfaction.

### ADLs

Very few studies have explored the impact of physical impairments in relation to functional independence. People with ARSACS aged 40 and over show restriction in their functional independence when compared to reference values and younger people. These restrictions are highly variable according to the diversity of clinical pictures seen in ARSACS [47]. Additionally, people who use wheelchairs or walking aids show a lower level of participation [46, 48, 49], the same for people aged 50 and older [19]. Affected people can also present slowness in performing daily activities [103]. Similar to physical activities, people with ARSACS involved in the study experience several difficulties performing ADLs, which interferes with their functional autonomy (7/12). These difficulties are related to their physical limitations, but they cannot identify specific causes. It seems that disabilities lead to a progressive loss of independence, which is slow and variable. The extent of the loss of independence begins with a slowness of execution toward more severe difficulties, even incapacities. Among the difficulties, people mention limitations related to household, cooking, and hygiene care. They have to develop a range of coping strategies to deal with everyday life, such as the use of technical or human aid.

## Mental health

The second component of the PROMIS conceptual framework is mental health, which includes affect, behavior, and cognition. The affect subcomponent is poorly documented in the literature and covers anxiety, depression, frustration, and the negative psychosocial impact of illness.

### Anxiety

Only one study reports the case of two persons affected who experience psychiatric disorders that involve anxiety and other psychiatric symptoms [85]. Additionally, a thesis reported that 49% of the 30 participants with ARSACS reported emotional distress demonstrated by anxiety [44]. In the same way, participants (2/12) of the study note anxiety, as they report feeling stressed for everything or being anxious when faced with the uncertain nature of the course of the disease.

### Depression

Two case studies discuss the case of a person with ARSACS who suffered from severe depression [85, 103]. The same thesis seen before reported that 10 to 26% of the participants showed depression symptoms [44]. In the sample, 4/12 participants noted variable depression-related manifestations, from transient sadness (in particular when thinking about the disease and its evolution) to the observation of more severe symptoms by the interwiewer (e.g., cries during the interview, avoid the question).

Frustration Except for the same case study that reports aggressive behavior with other psychiatric symptoms [85], there is no mention of frustration in the literature. However, half of the participants experienced manifestations of frustration in one way or another. The frustration is caused by everyday obstacles encountered (e.g., difficulty with employment, difficulty accessing services), the loss of independence, the feeling of loss of control, the need to constantly adapt to new limitations and the constant grievances caused by the progression of disease, not being able to help others, and not being able to do the same things as other people of their age. These frustrations can lead to feelings of sadness, culpability (e.g., when getting angry with relatives), and mood swings.

Negative psychosocial impact of illness. One thesis reports an elevated social desirability that can explain the desire for people with ARSACS to give a more positive self-image [44]. For two participants in the qualitative study, the perceived negative judgment of others can affect their self-image. The relatively early onset of the loss of mobility leads to the necessity of using a walking aid that can make them feel that they are older or disabled.

### Cognition

Some studies show difficulties with cognitive functions in ARSACS. Only one study reported no evidence of intellectual involvement [109], and some showed mild intellectual disability, mental retardation, or IQ below average [6, 13, 14, 20, 23, 36, 57, 66, 71, 85, 91–93, 103, 105, 106, 112, 121, 128, 134, 144]. Some pathological personality traits can also be encountered, such as mental rigidity and a poor degree of openness to experience [44]. People in the study do not recon cognitive dysfunction in terms of intellectual disability; instead, they do remark difficulties related to cognitive abilities. Some of them have noted variable decreases in their memory (e.g., forgetting names, appointments, memories), an increased time required to learn new things, difficulties concentrating (sometimes related to a deficit attention disorder) and some difficulties in analyzing complex situations. These difficulties are noted in the literature, as some studies report impaired memory [21, 63, 69], concentration problems [21], learning difficulties [20, 38, 79, 106], and other cognitive dysfunctions [34, 44, 62, 131].

## Social health

The third component of the PROMIS framework is social health, which has two subcomponents: social relationships and social roles and activities.

### Social relationships

Only one thesis reports features about social relationships in ARSACS. This study demonstrates that people affected feel less adapted in familial relationships in correlation with dysfunctional personality traits (nevrosism and dependant personality) [44]. In the qualitative study, some participants (2/12) felt like other people did not understand their condition, which can lead to situations such as intimidation or isolation. They also experienced difficulties with romantic relationships (5/12), as they found it difficult to find and keep a partner.

### Social roles

Social roles include capacities and satisfaction related to work, study, and parenthood. For the work subdomain, a qualitative study shows that people with ARSACS reported difficulties obtaining and, to a larger extent, keeping a job. These difficulties can partly be explained by the progression of physical limitations and cognitive rigidity [130]. They can lead to the need for a physically nondemanding job in an adapted workplace [134] or to be unemployed [19]. Another study reported that men with ARSACS have poorer social adaptation to work than the general population [44]. All of the participants with ARSACS in the qualitative study described dissatisfaction or limitations to work (pass or present): no longer being able to practice one's job due to limitations, reorientation following the progression of the limitations, difficulty or inability to work the required number of hours, dangerousness (risk of falling), dismissal, orientation difficulty, premature retirement, and difficulties in balancing work, family and ADLs. Participants also have to live with feeling of grief for not being able to work, perception that employers lack openness, perception of bullying in the workplace, frustration, feeling of injustice, and financial impact for not having a job.

Some studies documented the presence of difficulty at school that can be related to intellectual disability [66, 105], mild to moderate learning difficulty [20, 38, 56, 106] or the need for adaptations [119]. In the qualitative study, five participants explained that they had experienced difficulties at school because of their physical limitations and needed special adaptations (e.g., access to a portable computer, more time to do exams). During adulthood schooling, the difficulties are more related to maintaining a balance between study, work and ADLs.

Unsurprisingly, there is no literature about parenthood and ARSACS. In the sample, only two participants were parents. They describe some difficulties as they cannot perform all activities they want to do with children and can encounter some barriers (e.g., difficulty to lift the baby, risk of failing with the baby).

### Social activities

Very few studies have considered social activities. In terms of social participation, it seems that older people with ARSACS and those who use a wheelchair or walking aid have a lower level of social participation [46, 48,49]. Additionally, social adaptation regarding social life and leisures is lower for women with ARSACS [44]. In the sample, nine participants experienced dissatisfaction and difficulties related to the realization of their social activities. The physical manifestations and trouble in managing energy can limit the choice of activities (e.g., more sedentary activities, not being able to go to the same places as friends, adapted activities).

## Results part 2: comparison between ARSACS and other ARCA

For the discussion groups, group 1 was carried out in June 2019 and lasted approximately 109 min, while group 2 was realized in October 2019 and lasted approximately 144 min. People in the two groups were affected by one of these ataxias: Ataxia with oculomotor apraxia type 2 (AOA2), autosomal recessive spinocerebellar ataxia-8 (SCAR8) or Friedreich’s ataxia (FA). The characteristics of the groups’ participants are shown in Table 4.

**Table 4:** Characteristics of the study population for the discussion groups (n = 2, 8 participants)

Characteristics	Total group	Total group 1	Total group 2
Age			
Mean	43.8	49	40.6
Range	23–59	36–58	23–59
Sex (n)			
Female	3	0	3
Male	5	3	2
Diagnosis			
FA	3	0	3
SCAR8	4	2	2
AOA2	1	1	0

The aim of the discussion groups was to qualitatively compare autoreported manifestations of ARSACS with other types of ARCA. In general, manifestations, especially impacts of the disease, are similar. However, foot deformities seem to be more important for other types of ARCA; some participants described swollen and painful feet, heavy feet, or cold feet. Another dissimilarity between ARSACS and some other types of ARCA concerns eye functions. While people with ARSACS describe no particular problem with their vision, those with other types can experience diplopia that can hinder their daily life, such as driving and reading.

## Discussion

This article briefly summarizes the results of the first qualitative study in ARSACS patients documenting the manifestations and impacts of the disease according to affected persons. Participants reported impacts of the disease in the three components of the PROMIS conceptual framework: physical health, social health, and mental health. As we can predicted, physical health is the component that is the most studied. However, some symptoms and their impacts are scarcely or not documented in the literature. It’s the case with fatigue. Fatigue and energy management are symptoms that can have variable impacts on daily living. In a qualitative study of patients’ experience of ataxia (idiopathic or inherited), fatigue was reported as an issue by one-third of the participants [31]. In Friedreich ataxia (FA), fatigue is viewed as a factor that impacts sexual function [29]. The item *Loss of energy* of the Beck Depression Inventory is also significantly higher than the values for the general population and is most frequently endorsed by the participants [90].

All the affects included in the mental health component are poorly documented in the literature in ARSACS and in other forms of recessive ataxia. However, these negative feelings, such as depression, anxiety and frustration, can be experienced by a large proportion of people affected. A study that documented depressive symptoms in FA shows that the depression score was significantly higher than the mean score in the general population. The same study indicated a positive correlation between depression score and disease severity [90].

Among the PROMIS framework, social health and its related concepts are the less documented component in ARSACS. This study shows many impacts of the disease related to social roles and activities. The physical manifestations of the disease interfere with the perceived realization of social roles, such as employment and socialization. In a study examining the impact of FA on quality of life (QOL), Wilson et al. [142] indicated that social function is one of the mental dimensions of QOL most affected. Additionally, the presence of social support and interaction was associated with higher perceived physical QOL. In a qualitative study on symptoms of ataxia, the results indicate that people affected largely staying at home because of their mobility issues and fear of the negative judgment of others. They also described negative effects on employment (loss of job) and financial struggles [31].

The results of this study will serve as base point to develop a PRO measurement in recessive ataxia. In the literature, we found two PRO related to our findings one with population with any ataxia [118], and one for FA [24]. Both used the insight of people affected to identified manifestations and impacts of the disease, but none used patient-oriented research design. The involvement of patient as partner was a key feature to really understand the lived experience of the affected persons. The first PRO identified items by an online survey and the second performed in-depth semistructured interviews. In addition to the physical manifestations more documented in literature, they identified mental and social impacts of the disease. Social impacts include isolation and difficulty performing social roles like work or parenting. Mental impacts include different negative affects like frustration and depressive symptoms. This points out the necessity to gathered people affected perspective when developing PRO, to capture impacts that are not documented in more traditional ways.The principal strength of this study is to be the first qualitative study that documented symptoms and manifestations of ARSACS by the people affected. The perception of the people directly concerned by the disease gives a unique point of view, and the qualitative design brings much richness to the results. These results, compared to the data of the literature, bring a new comprehensive portrait of ARSAC. Other strengths include the use of several data collection methods (interviews, discussion groups, literature) for the triangulation of the data and the use of a conceptual framework and blueprint, which increase the credibility of the results. Limits include a small sample for the individual interviews that limit their generalization. On the other hand, the use of a saturation table ensures the saturation of the data, as it provides some evidence that the sample was sufficient to capture the complete experience of people affected. Another limitation is the fact that the study took place in a homogeneous francophone population in Québec. Although this is where most people with ARSACS are found worldwide, their experience can be influenced by their environment. The impact of this is limited by the literature review that includes cohorts in different parts of the world.

## Conclusion

This study provides a complete portrait of the manifestations and impacts of the disease according to the people affected and the literature. It contributes to partially filling the gap in knowledge regarding mental and social health. In the future, more research will be needed to properly document these components.

However, most of this study will serve as a basis for the development of a new patient-reported outcome measurement. This tool will aim to capture the entire experience of people affected with ARSACS and other forms of recessive ataxias. It can be used for clinical purposes and to measure the effects of treatments during therapeutic trials.

Additional references Table 3 [17].

## Data Availability

Please contact author for data requests.

## Abbreviations

ARCA: Autosomal recessive cerebellar ataxias
FA: Friedreich ataxia
ARSACS: Autosomal recessive spastic ataxia of Charlevoix–Saguenay
FDA: Food and drug administration
PRO: Patient-reported outcome
PROMIS: Patient-reported outcomes measurement information system
CIUSSS-SLSJ: Centre intégré universitaire de santé et de services sociaux du Saguenay–Lac-Saint-Jean
ADL: Activities of daily living
AOA2: Ataxia with oculomotor apraxia type 2
SCAR8: Autosomal recessive spinocerebellar ataxia-8
QOL: Quality of life

## References

[1] Abkur T, Vijayakumar K, Churchill AJ, Stevens J (2020). Clinical reasoning: complex ataxia: unpicking the threads. Neurology.

[2] Agarwal A, Garg D, Kharat A, Qavi A (2020). Autosomal recessive spastic ataxia of Charlevoix–Saguenay (ARSACS): case report of a novel nonsense mutation in the SACS gene. Ann Indian Acad Neurol.

[3] Agarwal PA, Ate-Upasani P, Ramprasad VL (2017). Autosomal recessive spastic ataxia of Charlevoix–Saguenay (ARSACS)-first report of clinical and imaging features from India, and a novel SACS gene duplication. Mov Disord Clin Pract.

[4] Aida I, Ozawa T, Fujinaka H, Goto K, Ohta K, Nakajima T (2021). Autosomal recessive spastic ataxia of Charlevoix–Saguenay without spasticity. Intern Med.

[5] Al-Ajmi A, Shamsah S, Janicijevic A, Williams M, Al-Mulla F (2020). Novel frameshift mutation in the SACS gene causing spastic ataxia of Charlevoix–Saguenay in a consanguineous family from the Arabian Peninsula: a case report and review of literature. World J Clin Cases.

[6] Ali Z, Klar J, Jameel M, Khan K, Fatima A, Raininko R (2016). Novel SACS mutations associated with intellectual disability, epilepsy and widespread supratentorial abnormalities. J Neurol Sci.

[7] Anheim M, Chaigne D, Fleury M, Santorelli FM, De Seze J, Durr A (2008). Autosomal recessive spastic ataxia of Charlevoix–Saguenay: study of a family and review of the literature. Rev Neurol (Paris).

[8] Anheim M, Fleury M, Monga B, Laugel V, Chaigne D, Rodier G (2010). Epidemiological, clinical, paraclinical and molecular study of a cohort of 102 patients affected with autosomal recessive progressive cerebellar ataxia from Alsace, Eastern France: implications for clinical management. Neurogenetics.

[9] Baets J, Deconinck T, Smets K, Goossens D, Van den Bergh P, Dahan K (2010). Mutations in SACS cause atypical and late-onset forms of ARSACS. Neurology.

[10] Blumkin L, Bradshaw T, Michelson M, Kopler T, Dahari D, Lerman-Sagie T (2015). Molecular and functional studies of retinal degeneration as a clinical presentation of SACS-related disorder. Eur J Paediatr Neurol.

[11] Borruat F-X, Holder GE, Bremner F (2017). Inner retinal dysfunction in the autosomal recessive spastic ataxia of Charlevoix–Saguenay. Front Neurol.

[12] Bouchard JP. Recessive spastic ataxia of Charlevoix–Saguenay. In: Vinken P, Bruyn G, Klawans HL, de Jong J, editors. Handbook of clinical neurology. Hereditary neuropathies and spinocerebellar atrophies (Vol. 16(60)). Amsterdam: Elsevier Science Pub.; 1991. p. 451–9.

[13] Bouchard JP, Barbeau A, Bouchard R, Bouchard RW (1978). Autosomal recessive spastic ataxia of Charlevoix–Saguenay. Can J Neurol Sci.

[14] Bouchard JP, Barbeau A, Bouchard R, Bouchard RW (1979). Electromyography and nerve conduction studies in Friedreich's ataxia and autosomal recessive spastic ataxia of Charlevoix–Saguenay (ARSACS). Can J Neurol Sci.

[15] Bouchard JP, Richter A, Mathieu J, Brunet D, Hudson TJ, Morgan K, Melançon SB (1998). Autosomal recessive spastic ataxia of Charlevoix–Saguenay. Neuromuscul Disord.

[16] Bouchard M, Langlois G (1999). Orthopedic management in autosomal recessive spastic ataxia of Charlevoix–Saguenay. Can J Surg.

[17] Bouhlal Y, Amouri R, El Euch-Fayeche G, Hentati F (2011). Autosomal recessive spastic ataxia of Charlevoix–Saguenay: an overview. Parkinsonism Relat Disord.

[18] Bouhlal Y, El Euch-Fayeche G, Hentati F, Amouri R (2009). A novel SACS gene mutation in a Tunisian family. J Mol Neurosci.

[19] Bourassa J, Routhier F, Gagnon C, Rahn C, Hébert LJ, St-Gelais R (2020). Wheelchair mobility, motor performance and participation of adult wheelchair users with ARSACS: a cross-sectional study. Disabil Rehabil Assist Technol.

[20] Breckpot J, Takiyama Y, Thienpont B, Van Vooren S, Vermeesch JR, Ortibus E, Devriendt K (2008). A novel genomic disorder: a deletion of the SACS gene leading to spastic ataxia of Charlevoix–Saguenay. Eur J Hum Genet.

[21] Briand MM, Rodrigue X, Lessard I, Mathieu J, Brais B, Côté I, Gagnon C (2019). Expanding the clinical description of autosomal recessive spastic ataxia of Charlevoix–Saguenay. J Neurol Sci.

[22] Burguêz D, Oliveira CM, Rockenbach M, Fussiger H, Vedolin LM, Winckler PB (2017). Autosomal recessive spastic ataxia of Charlevoix–Saguenay: a family report from South Brazil. Arq Neuropsiquiatr.

[23] Çakar A, İnci M, Özdağ Acarlı AN, Çomu S, Candayan A, Battaloğlu E (2022). Phenotypical spectrum of SACS variants: neuromuscular perspective of a complex neurodegenerative disorder. Acta Neurol Scand.

[24] Cano SJ, Riazi A, Schapira AH, Cooper JM, Hobart JC (2009). Friedreich's ataxia impact scale: a new measure striving to provide the flexibility required by today's studies. Mov Disord.

[25] Cappelleri JC, Zou KH, Bushmakin AG, Alvir JMJ, Alemayehu D, Symonds T (2014). Patient-reported outcomes: measurement, implementation and interpretation.

[26] Cella D, Yount S, Rothrock N, Gershon R, Cook K, Reeve B (2007). The patient-reported outcomes measurement information system (PROMIS): progress of an NIH Roadmap cooperative group during its first two years. Med Care.

[27] Chen Y, Cen Z, Zheng X, Chen S, Xie F, Luo W (2021). Novel compound heterozygous SACS mutations in a case with a spasticity-lacking phenotype of Sacsin-related ataxia. Neurol India.

[28] Cho H, Lyoo CH, Park SE, Seo Y, Han SH, Han J (2021). Optical coherence tomography findings facilitate the diagnosis of autosomal recessive spastic ataxia of Charlevoix–Saguenay. Korean J Ophthalmol.

[29] Corben LA, Hermans MM, Marks A, Crowe LM, Delatycki MB (2021). Sexual function, intimate relationships and Friedreich ataxia. J Neurol.

[30] Criscuolo C, Procaccini C, Meschini MC, Cianflone A, Carbone R, Doccini S (2015). Powerhouse failure and oxidative damage in autosomal recessive spastic ataxia of Charlevoix–Saguenay. J Neurol.

[31] Daker-White G, Kingston H, Payne K, Greenfield J, Ealing J, Sanders C (2015). You don't get told anything, they don't do anything and nothing changes. Medicine as a resource and constraint in progressive ataxia. Health Expect.

[32] De Braekeleer M, Giasson F, Mathieu J, Roy M, Bouchard JP, Morgan K (1993). Genetic epidemiology of autosomal recessive spastic ataxia of Charlevoix–Saguenay in Northeastern Quebec. Genet Epidemiol.

[33] de Wit MP, Berlo SE, Aanerud GJ, Aletaha D, Bijlsma JW, Croucher L (2011). European league against rheumatism recommendations for the inclusion of patient representatives in scientific projects. Ann Rheum Dis.

[34] Desserre J, Devos D, Sautière BG, Debruyne P, Santorelli FM, Vuillaume I, Defoort-Dhellemmes S (2011). Thickening of peripapillar retinal fibers for the diagnosis of autosomal recessive spastic ataxia of Charlevoix–Saguenay. Cerebellum.

[35] Dionne J, Wright G, Barber H, Bouchard R, Bouchard JP (1979). Oculomotor and vestibular findings in autosomal recessive spastic ataxia of Charlevoix–Saguenay. Can J Neurol Sci.

[36] Dougherty SC, Harper A, Al Saif H, Vorona G, Haines SR (2018). A chromosomal deletion and new Frameshift mutation cause ARSACS in an African-American. Front Neurol.

[37] Douglas VP, Douglas KAA, Miller JB, Gaier ED (2021). Absent Foveal avascular zone in autosomal recessive spastic ataxia of Charlevoix–Saguenay. J Neuroophthalmol.

[38] Duquette A, Brais B, Bouchard JP, Mathieu J (2013). Clinical presentation and early evolution of spastic ataxia of Charlevoix–Saguenay. Mov Disord.

[39] Dziewulska D (2020). Diplomyelia in a patient with a clinical suspicion of autosomal recessive spastic ataxia of Charlevoix–Saguenay type (ARSACS). Folia Neuropathol.

[40] El Euch-Fayache G, Lalani I, Amouri R, Turki I, Ouahchi K, Hung WY (2003). Phenotypic features and genetic findings in sacsin-related autosomal recessive ataxia in Tunisia. Arch Neurol.

[41] Engert JC, Berube P, Mercier J, Doré C, Lepage P, Ge B (2000). ARSACS, a spastic ataxia common in northeastern Quebec, is caused by mutations in a new gene encoding an 11.5-kb ORF. Nat Genet.

[42] Food and Drug Administration. Guidance for industry: patient-reported outcome measures: use in medical product development to support labeling claims. Rockville, MD: Department of Health and Human Services, Food and Drug Administration, Center for Drug Evaluation and Research; 2009. p. 39.

[43] Food and Drug Administration. Roadmap to patient-focused outcome measurement in clinical trials. Silver Spring, MD: Department of Health and Human Services, Food and Drug Administration; 2013.

[44] Forgue G, Bouchard J, Gallais B. Description des traits de personnalité et de l'adaptation sociale chez des personnes atteintes d'ataxie récessive spastique de Charlevoix–Saguenay. (Essai Doctoral), Université du Québec à Chicoutimi. 2019. Retrieved from https://constellation.uqac.ca/5178/1/Forgues_uqac_0862D_10571.pdf

[45] Francis JJ, Johnston M, Robertson C, Glidewell L, Entwistle V, Eccles MP, Grimshaw JM (2010). What is an adequate sample size? Operationalising data saturation for theory-based interview studies. Psychol Health.

[46] Gagnon C, Brais B, Lessard I, Lavoie C, Côté I, Mathieu J (2018). From motor performance to participation: a quantitative descriptive study in adults with autosomal recessive spastic ataxia of Charlevoix–Saguenay. Orphanet J Rare Dis.

[47] Gagnon C, Desrosiers J, Mathieu J (2004). Autosomal recessive spastic ataxia of Charlevoix–Saguenay: upper extremity aptitudes, functional independence and social participation. Int J Rehabil Res.

[48] Gagnon C, Lessard I, Brais B, Cote I, Lavoie C, Synofzik M, Mathieu J (2018). Validity and reliability of outcome measures assessing dexterity, coordination, and upper limb strength in autosomal recessive spastic ataxia of Charlevoix–Saguenay. Arch Phys Med Rehabil.

[49] Gagnon C, Lessard I, Lavoie C, Côté I, St-Gelais R, Mathieu J, Brais B (2018). An exploratory natural history of ataxia of Charlevoix–Saguenay: a 2-year follow-up. Neurology.

[50] Gallagher F, Corbière M, Larivière N (2014). La recherche descriptive interprétative : Description des besoins psychosociaux de femmes à la suite d’un résultat anormal à la mammographie de dépistage du cancer du sein. Méthodes qualitatives, quantitatives et mixtes: dans la recherche en sciences humaines, sociales et de la santé.

[51] Garcia A, Criscuolo C, de Michele G, Berciano J (2008). Neurophysiological study in a Spanish family with recessive spastic ataxia of Charlevoix–Saguenay. Muscle Nerve.

[52] Gazulla J, Benavente I, Vela AC, Marín MA, Pablo LE, Tessa A (2012). New findings in the ataxia of Charlevoix–Saguenay. J Neurol.

[53] Gazulla J, Mayayo-Sinues E, Benavente I, Modrego PJ, Berciano J (2014). Ataxia of Charlevoix–Saguenay: MR and clinical results in lower-limb musculature. Can J Neurol Sci.

[54] Gazulla J, Vela AC, Marin MA, Pablo L, Santorelli FM, Benavente I (2011). Is the ataxia of Charlevoix–Saguenay a developmental disease?. Med Hypotheses.

[55] Gregianin E, Vazza G, Scaramel E, Boaretto F, Vettori A, Leonardi E (2013). A novel SACS mutation results in non-ataxic spastic paraplegia and peripheral neuropathy. Eur J Neurol.

[56] Grieco GS, Malandrini A, Comanducci G, Leuzzi V, Valoppi M, Tessa A (2004). Novel SACS mutations in autosomal recessive spastic ataxia of Charlevoix–Saguenay type. Neurology.

[57] Gücüyener K, Ozgül K, Paternotte C, Erdem H, Prud'homme JF, Ozgüç M, Topaloğlu H (2001). Autosomal recessive spastic ataxia of Charlevoix–Saguenay in two unrelated Turkish families. Neuropediatrics.

[58] Guest G, Bunce A, Johnson L (2006). How many interviews are enough? An experiment with data saturation and variability. Field Methods.

[59] Habibzadeh P, Tabatabaei Z, Inaloo S, Nashatizadeh MM, Synofzik M, Ostovan VR, Faghihi MA (2020). Case report: expanding the genetic and phenotypic spectrum of autosomal recessive spastic ataxia of Charlevoix–Saguenay. Front Genet.

[60] Haga R, Miki Y, Funamizu Y, Kon T, Suzuki C, Ueno T (2012). Novel compound heterozygous mutations of the SACS gene in autosomal recessive spastic ataxia of Charlevoix–Saguenay. Clin Neurol Neurosurg.

[61] Halcomb EJ, Davidson PM (2006). Is verbatim transcription of interview data always necessary?. Appl Nurs Res.

[62] Hamza W, Ali Pacha L, Hamadouche T, Muller J, Drouot N, Ferrat F (2015). Molecular and clinical study of a cohort of 110 Algerian patients with autosomal recessive ataxia. BMC Med Genet.

[63] Hara K, Onodera O, Endo M, Kondo H, Shiota H, Miki K (2005). Sacsin-related autosomal recessive ataxia without prominent retinal myelinated fibers in Japan. Mov Disord.

[64] Incecik F, Hergüner OM, Bisgin A (2018). Autosomal-recessive spastic ataxia of Charlevoix–Saguenay: a Turkish child. J Pediatr Neurosci.

[65] Jacobi H, du Montcel ST, Bauer P, Giunti P, Cook A, Labrum R (2018). Long-term evolution of patient-reported outcome measures in spinocerebellar ataxias. J Neurol.

[66] Kamada S, Okawa S, Imota T, Sugawara M, Toyoshima I (2008). Autosomal recessive spastic ataxia of Charlevoix–Saguenay (ARSACS): novel compound heterozygous mutations in the SACS gene. J Neurol.

[67] Karuvath RH, Patwari S, Chadaga H (2021). Case 293: autosomal recessive spastic ataxia of Charlevoix–Saguenay. Radiology.

[68] Kerr C, Nixon A, Wild D (2010). Assessing and demonstrating data saturation in qualitative inquiry supporting patient-reported outcomes research. Expert Rev Pharmacoecon Outcomes Res.

[69] Krygier M, Konkel A, Schinwelski M, Rydzanicz M, Walczak A, Sildatke-Bauer M (2017). Autosomal recessive spastic ataxia of Charlevoix–Saguenay (ARSACS): a polish family with novel SACS mutations. Neurol Neurochir Pol.

[70] Krygier M, Kwarciany M, Wasilewska K, Pienkowski VM, Krawczyńska N, Zielonka D, Rydzanicz M (2019). A study in a Polish ataxia cohort indicates genetic heterogeneity and points to MTCL1 as a novel candidate gene. Clin Genet..

[71] Kuchay RAH, Mir YR, Zeng X, Hassan A, Musarrat J, Parwez I (2019). ARSACS as a worldwide disease: novel SACS mutations identified in a consanguineous family from the remote tribal Jammu and Kashmir region in India. Cerebellum.

[72] Kwon KY, Huh K, Eun BL, Yoo HW, Kamsteeg EJ, Scheffer H, Koh SB (2015). A probable Korean case of autosomal recessive spastic ataxia of Charlevoix–Saguenay. Can J Neurol Sci.

[73] Larivière R, Gaudet R, Gentil BJ, Girard M, Conte TC, Minotti S (2014). Sacs knockout mice present pathophysiological defects underlying autosomal recessive spastic ataxia of Charlevoix–Saguenay. Hum Mol Genet.

[74] Leavitt JA, Singer W, Brown WL, Pulido JS, Brodsky MC (2014). Retinal and pontine striations: neurodiagnostic signs of autosomal recessive spastic ataxia of Charlevoix–Saguenay. J Neuroophthalmol.

[75] Lessard I, Brais B, Côté I, Lavoie C, Synofzik M, Mathieu J, Gagnon C (2018). Assessing mobility and balance in autosomal recessive spastic ataxia of Charlevoix–Saguenay population: validity and reliability of four outcome measures. J Neurol Sci.

[76] Lessard I, St-Gelais R, Hébert LJ, Côté I, Mathieu J, Brais B, Gagnon C (2021). Functional mobility in walking adult population with ataxia of Charlevoix–Saguenay. Orphanet J Rare Dis.

[77] Liew WK, Ben-Omran T, Darras BT, Prabhu SP, De Vivo DC, Vatta M (2013). Clinical application of whole-exome sequencing: a novel autosomal recessive spastic ataxia of Charlevoix–Saguenay sequence variation in a child with ataxia. JAMA Neurol.

[78] Liu L, Li XB, Zi XH, Shen L, Hu Zh, M., Huang Sh, X., (2016). A novel hemizygous SACS mutation identified by whole exome sequencing and SNP array analysis in a Chinese ARSACS patient. J Neurol Sci.

[79] Lu Q, Shang L, Tian WT, Cao L, Zhang X, Liu Q (2020). Complicated paroxysmal kinesigenic dyskinesia associated with SACS mutations. Ann Transl Med.

[80] Martin MH, Bouchard JP, Sylvain M, St-Onge O, Truchon S (2007). Autosomal recessive spastic ataxia of Charlevoix–Saguenay: a report of MR imaging in 5 patients. AJNR Am J Neuroradiol.

[81] Masciullo M, Modoni A, Tessa A, Santorelli F, Rizzo V, D'Amico G (2012). Novel SACS mutations in two unrelated Italian patients with spastic ataxia: clinico-diagnostic characterization and results of serial brain MRI studies. Eur J Neurol.

[82] Masciullo M, Silvestri G, Modoni A, Tessa A, Bianchi ML, Santorelli FM (2014). Do not jump to easy conclusions! Lessons from pitfall in the molecular diagnosis of ARSACS. Clin Genet.

[83] McKenzie E, Sharma P, Parboosingh J, Suchowersky O, Consortium FC (2014). Novel SACS mutation deviates from the French Canadian ARSACS phenotype. Can J Neurol Sci.

[84] McMillan HJ, Carter MT, Jacob PJ, Laffan EE, O'Connor MD, Boycott KM (2009). Homozygous contiguous gene deletion of 13q12 causing LGMD2C and ARSACS in the same patient. Muscle Nerve.

[85] Mignarri A, Tessa A, Carluccio MA, Rufa A, Storti E, Bonelli G (2014). Cerebellum and neuropsychiatric disorders: insights from ARSACS. Neurol Sci.

[86] Miles MB, Huberman AM, Sadana J (2014). Qualitative data analysis: a method sourcebook.

[87] Miyatake S, Miyake N, Doi H, Saitsu H, Ogata K, Kawai M, Matsumoto N (2012). A novel SACS mutation in an atypical case with autosomal recessive spastic ataxia of Charlevoix–Saguenay (ARSACS). Intern Med.

[88] Muslemani S, Lessard I, Lavoie C, Cote I, Brais B, Mathieu J, Gagnon C. Exploratory study of participation and functional independence in adults with autosomal recessive spastic ataxia of Charlevoix–Saguenay. Can J Occup Ther.; Submitted. 10.1177/00084174221088417 PMC9511234 35469466

[89] Narayanan V, Rice SG, Olfers SS, Sivakumar K (2011). Autosomal recessive spastic ataxia of Charlevoix–Saguenay: compound heterozygotes for nonsense mutations of the SACS gene. J Child Neurol.

[90] Nieto A, Hernández-Torres A, Pérez-Flores J, Montón F (2018). Depressive symptoms in Friedreich ataxia. Int J Clin Health Psychol.

[91] Ogawa T, Takiyama Y, Sakoe K, Mori K, Namekawa M, Shimazaki H (2004). Identification of a SACS gene missense mutation in ARSACS. Neurology.

[92] Oguz KK, Haliloglu G, Temucin C, Gocmen R, Has AC, Doerschner K (2013). Assessment of whole-brain white matter by DTI in autosomal recessive spastic ataxia of Charlevoix–Saguenay. AJNR Am J Neuroradiol.

[93] Okawa S, Sugawara M, Watanabe S, Imota T, Toyoshima I (2006). A novel sacsin mutation in a Japanese woman showing clinical uniformity of autosomal recessive spastic ataxia of Charlevoix–Saguenay. J Neurol Neurosurg Psychiatry.

[94] Ouyang Y, Segers K, Bouquiaux O, Wang FC, Janin N, Andris C (2008). Novel SACS mutation in a Belgian family with sacsin-related ataxia. J Neurol Sci.

[95] Ouyang Y, Takiyama Y, Sakoe K, Shimazaki H, Ogawa T, Nagano S (2006). Sacsin-related ataxia (ARSACS): expanding the genotype upstream from the gigantic exon. Neurology.

[96] Pablo LE, Garcia-Martin E, Gazulla J, Larrosa JM, Ferreras A, Santorelli FM (2011). Retinal nerve fiber hypertrophy in ataxia of Charlevoix–Saguenay patients. Mol Vis.

[97] Paillé P, Mucchielli A (2008). L'analyse qualitative en sciences humaines et sociales.

[98] Palau F, Espinós C (2006). Autosomal recessive cerebellar ataxias. Orphanet J Rare Dis.

[99] Palmio J, Kärppä M, Baumann P, Penttilä S, Moilanen J, Udd B (2016). Novel compound heterozygous mutation in SACS gene leads to a milder autosomal recessive spastic ataxia of Charlevoix–Saguenay, ARSACS, in a Finnish family. Clin Case Rep.

[100] Parkinson MH, Bartmann AP, Clayton LMS, Nethisinghe S, Pfundt R, Chapple JP (2018). Optical coherence tomography in autosomal recessive spastic ataxia of Charlevoix–Saguenay. Brain.

[101] Pedroso JL, Braga-Neto P, Abrahão A, Rivero RL, Abdalla C, Abdala N, Barsottini OG (2011). Autosomal recessive spastic ataxia of Charlevoix–Saguenay (ARSACS): typical clinical and neuroimaging features in a Brazilian family. Arq Neuropsiquiatr.

[102] Pensabene MC, Melis M, Corato L, Stefano CD, Pizzicannella G, Mondillo M (2020). Autosomal recessive spastic ataxia of Charlevoix–Saguenay: findings from MRI in two adult Italian siblings. Radiol Case Rep.

[103] Petrov I (2021). Novel mutation in SACS gene in a patient with autosomal recessive spastic ataxia Charlevoix–Saguenay. Mov Disord Clin Pract.

[104] Picher-Martel V, Dupre N (2018). Current and promising therapies in autosomal recessive ataxias. CNS Neurol Disord Drug Targets.

[105] Pilliod J, Moutton S, Lavie J, Maurat E, Hubert C, Bellance N (2015). New practical definitions for the diagnosis of autosomal recessive spastic ataxia of Charlevoix–Saguenay. Ann Neurol.

[106] Prodi E, Grisoli M, Panzeri M, Minati L, Fattori F, Erbetta A (2013). Supratentorial and pontine MRI abnormalities characterize recessive spastic ataxia of Charlevoix–Saguenay. A comprehensive study of an Italian series. Eur J Neurol.

[107] Rezende Filho FM, Parkinson MH, Pedroso JL, Poh R, Faber I, Lourenço CM (2019). Clinical, ophthalmological, imaging and genetic features in Brazilian patients with ARSACS. Parkinsonism Relat Disord.

[108] Rezende Filho FM, Pedroso JL, Barsottini OGP (2018). An Unusual Fundus Finding in a Teenage Girl. JAMA Neurol.

[109] Ricca I, Morani F, Bacci GM, Nesti C, Caputo R, Tessa A, Santorelli FM (2019). Clinical and molecular studies in two new cases of ARSACS. Neurogenetics.

[110] Ricca I, Tessa A, Trovato R, Bacci GM, Santorelli FM (2020). Docosahexaenoic acid in ARSACS: observations in two patients. BMC Neurol.

[111] Richards C, Bouchard JP, Bouchard R, Barbeau H (1980). A preliminary study of dynamic muscle function in hereditary ataxia. Can J Neurol Sci.

[112] Richter AM, Ozgul RK, Poisson VC, Topaloglu H (2004). Private SACS mutations in autosomal recessive spastic ataxia of Charlevoix–Saguenay (ARSACS) families from Turkey. Neurogenetics.

[113] Robitaille Y, Richter A, Mathieu J, Bouchard JP. ARSACS. Autosomal recessive spastic ataxia of Charlevoix–Saguenay. GeneReviews at GeneTests. 1997–2007. 2007, January 2009. Retrieved December, 2009, from http://www.genetests.org accessible en ligne : http://www.ncbi.nlm.nih.gov/bookshelf/br.fcgi?book=gene&part=arsacs

[114] Rothman M, Burke L, Erickson P, Leidy NK, Patrick DL, Petrie CD (2009). Use of existing patient-reported outcome (PRO) instruments and their modification: the ISPOR good research practices for evaluating and documenting content validity for the use of existing instruments and their modification PRO Task Force Report. Value Health.

[115] Saffie P, Kauffman MA, Fernandez JM, Acosta I, Espay AJ, de la Cerda A (2017). Teaching video neuroImages: spastic ataxia syndrome. Neurology.

[116] Sahin T, Karaarslan FT, Yilmaz R, Tekgül Ş, Başak AN, Akbostanci MC (2021). Two cases of early-onset autosomal recessive spastic ataxia of Charlevoix–Saguenay diagnosed in adulthood. Clin Neurol Neurosurg.

[117] Samanci B, Gokalp EE, Bilgic B, Gurvit H, Artan S, Hanagasi HA (2021). A novel SACS p.Pro4154GlnfsTer20 mutation in a family with autosomal recessive spastic ataxia of Charlevoix–Saguenay. Neurol Sci.

[118] Schmahmann JD, Pierce S, MacMore J, L'Italien GJ. Development and validation of a patient‐reported outcome measure of ataxia. Mov Disord. 2021. 10.1002/mds.28670 34115419

[119] Sheetal S, Kumar SA, Byju P (2020). SACS mutation-positive autosomal recessive spastic ataxia of Charlevoix Saguenay (ARSACS) from Kerala. Ann Indian Acad Neurol.

[120] Shimazaki H, Sakoe K, Niijima K, Nakano I, Takiyama Y (2007). An unusual case of a spasticity-lacking phenotype with a novel SACS mutation. J Neurol Sci.

[121] Shimazaki H, Takiyama Y, Honda J, Sakoe K, Namekawa M, Tsugawa J (2013). Middle cerebellar peduncles and Pontine T2 hypointensities in ARSACS. J Neuroimaging.

[122] Shimazaki H, Takiyama Y, Sakoe K, Ando Y, Nakano I (2005). A phenotype without spasticity in sacsin-related ataxia. Neurology.

[123] Srikajon J, Pitakpatapee Y, Limwongse C, Chirapapaisan N, Srivanitchapoom P (2020). Autosomal recessive spastic ataxia of Charlevoix–Saguenay (ARSACS) in a Thai patient: the classic clinical manifestations, funduscopic feature, and brain imaging findings with a novel mutation in the SACS gene. Tremor Other Hyperkinet Mov (NY).

[124] Stevens JC, Murphy SM, Davagnanam I, Phadke R, Anderson G, Nethisinghe S (2013). The ARSACS phenotype can include supranuclear gaze palsy and skin lipofuscin deposits. J Neurol Neurosurg Psychiatry.

[125] Streiner DL, Norman GR, Cairney J (2015). Health measurement scales: a practical guide to their development and use.

[126] Synofzik M, Németh AH (2018). Recessive ataxias. Handb Clin Neurol.

[127] Synofzik M, Soehn AS, Gburek-Augustat J, Schicks J, Karle KN, Schule R (2013). Autosomal recessive spastic ataxia of Charlevoix Saguenay (ARSACS): expanding the genetic, clinical and imaging spectrum. Orphanet J Rare Dis.

[128] Terracciano A, Casali C, Grieco GS, Orteschi D, Di Giandomenico S, Seminara L (2009). An inherited large-scale rearrangement in SACS associated with spastic ataxia and hearing loss. Neurogenetics.

[129] Terracciano A, Foulds NC, Ditchfield A, Bunyan DJ, Crolla JA, Huang S (2010). Pseudodominant inheritance of spastic ataxia of Charlevoix–Saguenay. Neurology.

[130] Tremblay M, Laberge L, Maltais D, Durand M-J, Chouinard M-C, Gagnon C. Accès et intégration en emploi chez les personnes atteintes d’ataxie récessive spastique de Charlevoix–Saguenay: le rôle de l’ergothérapeute. *ErgOThérapies.* 2020; 76.

[131] Tzoulis C, Johansson S, Haukanes BI, Boman H, Knappskog PM, Bindoff LA (2013). Novel SACS mutations identified by whole exome sequencing in a norwegian family with autosomal recessive spastic ataxia of Charlevoix–Saguenay. PLOS ONE.

[132] Van Damme P, Demaerel P, Spileers W, Robberecht W (2009). Autosomal recessive spastic ataxia of Charlevoix–Saguenay. Neurology.

[133] van Lint M, Hoornaert K, Ten Tusscher MPM (2016). Retinal nerve fiber layer thickening in ARSACS carriers. J Neurol Sci.

[134] Verhoeven WM, Egger JI, Ahmed AI, Kremer BP, Vermeer S, van de Warrenburg BP (2012). Cerebellar cognitive affective syndrome and autosomal recessive spastic ataxia of Charlevoix–Saguenay: a report of two male sibs. Psychopathology.

[135] Verhoeven WMA, Egger JIM, Ahmed AIA, Kremer BPH, Vermeer S, van Warrenburg BPC (2020). Clinical phenomena of Charlevoix–Saguenay ataxia in two adult brothers. Eur Psychiatry.

[136] Vermeer S, Meijer RP, Pijl BJ, Timmermans J, Cruysberg JR, Bos MM (2008). ARSACS in the Dutch population: a frequent cause of early-onset cerebellar ataxia. Neurogenetics.

[137] Vill K, Müller-Felber W, Gläser D, Kuhn M, Teusch V, Schreiber H (2018). SACS variants are a relevant cause of autosomal recessive hereditary motor and sensory neuropathy. Hum Genet.

[138] Vingolo EM, Di Fabio R, Salvatore S, Grieco G, Bertini E, Leuzzi V (2011). Myelinated retinal fibers in autosomal recessive spastic ataxia of Charlevoix–Saguenay. Eur J Neurol.

[139] Vogel AP, Rommel N, Oettinger A, Stoll LH, Kraus E-M, Gagnon C (2018). Coordination and timing deficits in speech and swallowing in autosomal recessive spastic ataxia of Charlevoix–Saguenay (ARSACS). J Neurol.

[140] Wagner F, Titelbaum DS, Engisch R, Coskun EK, Waugh JL (2019). Subtle imaging findings aid the diagnosis of adolescent hereditary spastic paraplegia and ataxia. Clin Neuroradiol.

[141] Wang Z, Song Y, Wang X, Li X, Xu F, Si L (2021). Autosomal recessive spastic ataxia of Charlevoix–Saguenay caused by novel mutations in SACS gene: a report of two Chinese families. Neurosci Lett.

[142] Wilson CL, Fahey MC, Corben LA, Collins VR, Churchyard AJ, Lamont PJ, Delatycki MB (2007). Quality of life in Friedreich ataxia: What clinical, social and demographic factors are important?. Eur J Neurol.

[143] Xiromerisiou G, Dadouli K, Marogianni C, Provatas A, Ntellas P, Rikos D (2020). A novel homozygous SACS mutation identified by whole exome sequencing-genotype phenotype correlations of all published cases. J Mol Neurosci.

[144] Yamamoto Y, Hiraoka K, Araki M, Nagano S, Shimazaki H, Takiyama Y, Sakoda S (2005). Novel compound heterozygous mutations in sacsin-related ataxia. J Neurol Sci.

[145] Yu-Wai-Man P, Pyle A, Griffin H, Santibanez-Korev M, Horvath R, Chinnery PF (2014). Abnormal retinal thickening is a common feature among patients with ARSACS-related phenotypes. Br J Ophthalmol.

